# Cost-effectiveness of the latent tuberculosis screening program for migrants in Stockholm Region

**DOI:** 10.1007/s10198-021-01265-5

**Published:** 2021-02-09

**Authors:** Jad Shedrawy, Charlotte Deogan, Joanna Nederby Öhd, Maria-Pia Hergens, Judith Bruchfeld, Jerker Jonsson, Andrew Siroka, Knut Lönnroth

**Affiliations:** 1 Department of Global Public Health, Karolinska Institutet, Stockholm, Sweden; 2 The Public Health Agency of Sweden, Stockholm, Sweden; 3 Department of Communicable Disease Control and Prevention, Stockholm County Council, Stockholm, Sweden; 4 Department of Infectious Diseases, Karolinska University Hospital, Stockholm, Sweden; 5 Division of Infectious Diseases, Department of Medicine Solna, Karolinska Institutet, Stockholm, Sweden; 6 World Health Organization, Geneva, Switzerland

**Keywords:** Latent tuberculosis, Cost-effectiveness, Screening, Migrants, i10, i14

## Abstract

**Introduction:**

The majority of tuberculosis (TB) cases in Sweden occur among migrants from endemic countries through activation of latent tuberculosis infection (LTBI). Sweden has LTBI-screening policies for migrants that have not been previously evaluated. This study aimed to assess the cost-effectiveness of the current screening strategy in Stockholm.

**Methods:**

A Markov model was developed to predict the costs and effects of the current LTBI-screening program compared to a scenario of no LTBI screening over a 50-year time horizon. Epidemiological and cost data were obtained from local sources when available. The primary outcomes were incremental cost-effectiveness ratio (ICER) in terms of societal cost per quality-adjusted life year (QALY).

**Results:**

Screening migrants in the age group 13–19 years had the lowest ICER, 300,082 Swedish Kronor (SEK)/QALY, which is considered cost-effective in Sweden. In the age group 20–34, ICER was 714,527 SEK/QALY (moderately cost-effectives) and in all age groups above 34 ICERs were above 1,000,000 SEK/QALY (not cost-effective). ICER decreased with increasing TB incidence in country of origin.

**Conclusion:**

Screening is cost-effective for young cohorts, mainly between 13 and 19, while cost-effectiveness in age group 20–34 years could be enhanced by focusing on migrants from highest incidence countries and/or by increasing the LTBI treatment initiation rate. Screening is not cost-effective in older cohorts regardless of the country of origin.

## Introduction

Tuberculosis (TB) is a global public health concern with about 10 million people falling sick and 1.4 million deaths worldwide annually [1]. Aside from the active form of the disease, an individual can have latent TB infection (LTBI), a latency state in which the person is infected but healthy, asymptomatic and non-infectious. LTBI can activate to active TB at any time [2]. It is estimated that around one-fourth of the world’s population have LTBI, of which about 10% activate to symptomatic disease at any point in life. The risk of activation is highest soon after infection and is elevated by comorbidities such as HIV, diabetes, undernourishment, chronic kidney diseases and immunocompromising treatments [3–6]. LTBI-screening tools as well as efficacious preventive treatments are available, and therefore, LTBI screening and management in risk groups is an important element of TB control [5, 7].

In many low-incidence countries, domestic transmission is low and incident TB cases tends to be dominated by activation among immigrants from high-incidence countries who have acquired LTBI outside the host country (in the home country or during transit) [8, 9]. LTBI screening and management in key populations such as migrants is, therefore, an important part of TB elimination strategies in many low-incidence countries [7]. However, there is a lack of evidence about the most effective and cost-effective screening strategies in terms of which migrants to screen (e.g., based on age and TB incidence in country of origin) and which screening algorithm to use [10, 11]. Consequently, there is large variation in TB/LTBI-screening policies for migrants globally and across European countries [9].

In Sweden, about 90% of the TB cases are among foreign born. Recent migrants from high-incidence countries are more likely to have had a recent contact with a TB case, and therefore, are the groups with highest TB incidence. Asylum seekers, quota refugees and some reunified family members are offered a post-arrival, voluntary, free-of-charge health examination (HE). All attending HE are screened for TB symptoms and TB exposure risk factors (having been in refugee camp or prison or having had recent contact with a case of active TB) for all. All individuals with a TB exposure risk factor or coming from a country with TB incidence higher than 100/100,000 are systematically offered LTBI testing with Interferon-Gamma Release Assays (IGRA) or Tuberculin Skin Test (TST). Chest X-ray (CXR) is done for all who have TB symptoms or are IGRA/TST positive [12, 13]. Preventive treatment after LTBI diagnosis (and exclusion of active TB) is offered depending on age and risk factors. The regional guidelines in Stockholm country recommend treatment for all persons below the age of 20, women with recent pregnancy and people with an immunosuppressing condition or treatment. Treatment for patients 20 years of age or older is recommended only if a risk factor for progression is present. IGRA is used almost exclusively in Stockholm. The most commonly prescribed treatment regimens in Stockholm are: 4-month daily rifampin or 3-month daily combination of isoniazid and rifampin [14].

Although in place since the 1990s, the Swedish migrant TB-screening strategy has never been evaluated in terms of its cost-effectiveness. Therefore, the aim of this study was to determine the cost-effectiveness of the current LTBI-screening strategy compared to a scenario of no screening program for subgroups based on age and country of origin.

## Materials and methods

### Study population

A cohort of all migrants in Stockholm region attending HE and being tested for LTBI between January 1st, 2015 and December 31st, 2018 was established through extracting eligible persons from a registry called VeraAsyl, which is based on data from the Swedish Migration Agency. The cohort consisted of 5470 screened individuals who could be followed through screening, CXR, referral and visit to specialist care, LTBI treatment initiation and completion through linkages between VeraAsyl and electronic medical records in primary (screening data) and secondary care (treatment data). Details of the screening algorithm, the cohort, the methodology for ascertaining completion of each step of the screening and treatment cascade are reported elsewhere [15]. There was a statistically significant increase in LTBI positivity in higher age groups and among people from countries with higher TB burden (> 200/100,000) [15]. The empirical values for the parameters used in the present model are listed in appendix 1 in the support material.

### Cost-effectiveness analysis overview

A societal-perspective Markov model with a 50-year time horizon was developed to assess incremental cost in relation to incremental improvements in long-term health outcomes—TB cases prevented and quality-adjusted life years (QALY) gained, respectively—for two scenarios (arms of comparisons): the current migrant LTBI-screening strategy as implemented in Stockholm 2015–2018 versus a hypothetical scenario of no systematic migrant LTBI screening and treatment.

The model was run for different age groups and groups by TB incidence in country of origin (using WHO national incidence estimates), with current screening vs. no screening as arms of comparison in all analyses. The reason for running the analysis by subgroups rather than for the whole cohort is that prevalence of LTBI correlate with both age and TB epidemiology in country of origin. Moreover, recommendations and practice concerning treatment indication for persons with LTBI varies by age, especially since older persons have a higher risk of adverse drug reactions and harm of preventive treatment may outweigh benefit. Assessing cost-effectiveness for specific subgroups can help identify if and how targeting of screening affects cost-effectiveness and hence inform strategy adjustments.

The methodology used in this study follows a theoretical framework that has developed through a systematic review of methods in published CEA studies on LTBI screening in migrants [16].

Local cost data and Health-Related Quality of Life (HRQoL) data were discounted by 3% per annum according to the current Swedish recommendations [17]. The results are presented in terms of incremental cost-effectiveness ratios (ICER): the incremental cost of achieving one additional QALY or one additional prevented case with screening vs. no screening for each subgroup.

The analysis adopted a societal perspective as recommended in Sweden by The Dental and Pharmaceutical Benefits Agency (TLV) [17]. This perspective implies considering the costs and effects for the society including costs (and savings) for the health care services, costs for the Migration Agency (which funds the HE), out-of-pocket payment by the patient, and the productivity loss due to disease [18].

ICERs were judged against the cost-effectiveness thresholds recommended by the National Board of Health and Welfare [19], which considers an intervention very cost-effective if the ICER is below 100,000 SEK/QALY, cost-effective if the ICER is between 100,000 and 500,000 SEK/QALY, moderately cost-effective if the ICER is between 500,000 and 1,000,000 SEK/QALY, and not cost-effective if the ICER is above 1,000,000 SEK/QALY.

### Decision analytic model design

**Fig. 1:**
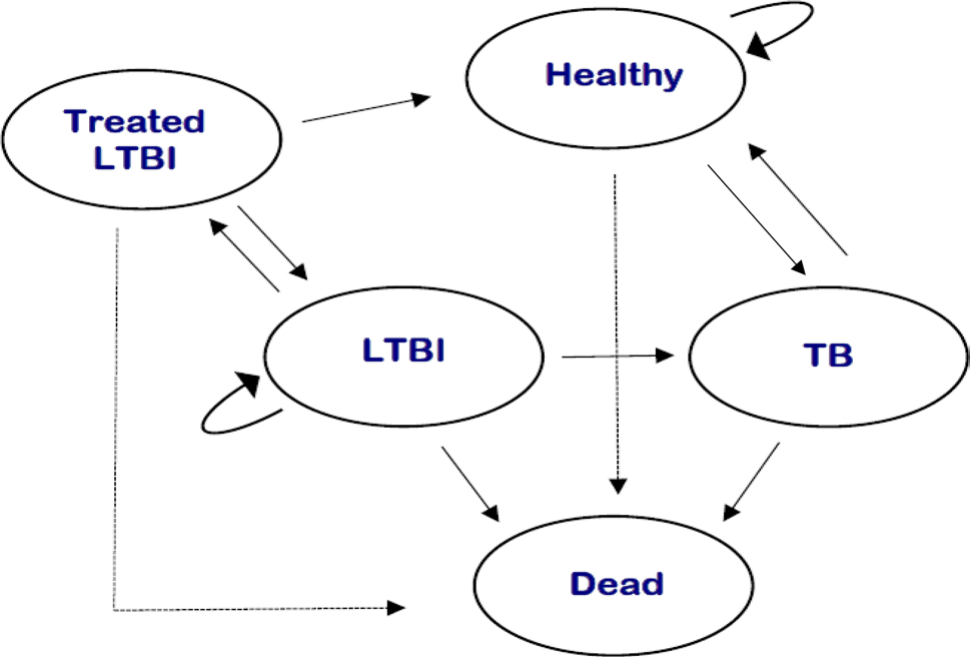
Simplified structure of the model representing all the health states and possible interactions

The Markov model structure was developed in Excel. Individuals could reside in one of five mutually exclusive health states (Fig. 1): (a) “Healthy” referring to a state of no TB and no LTBI; (b) “LTBI state” referring to undiagnosed LTBI or diagnosed but untreated or unsuccessfully treated LTBI; (c) “Treated LTBI state” referring to a successfully treated LTBI; (d) “Active TB state” referring to active TB disease; and (e) “Dead state” referring to death.

Movements between these were determined by probabilities that change according to age and the time of the cycle. Age and country of origin were the main determinant of a positive IGRA test, and age was the main determinants of initiating LTBI treatment after a positive IGRA as reported elsewhere [16]. The cycle length was chosen to be 6 months to accommodate for the most common treatment periods. To simplify the model and avoid having additional health states for active TB, the TB state was averaged to include sensitive TB (87%), mono-resistant TB (10%) and multi-drug resistant TB (MDR-TB) (3%), reflecting the epidemiological situation in this cohort.

### Sensitivity analyses

Epidemiological parameters that were not based on local empirical data—reactivation rate, death due to TB, treatment efficacy and secondary transmission—were varied separately within ranges shown in Table 1.

**Table 1:** Epidemiological parameters used in the model

Rate of activation from latent to active TB, per year	Model value	Ranges for univariate sensitivity analysis		References
		Lower value	Upper value	
First 2 years	0.025	0.01875	0.03125	[20, 21]
After 2 years, for life	0.001	0.00075	0.00125	
Treatment efficacy	0.775	0.6	0.9	[22]
Increased percentage of death due to TB	0.07	0.0525	0.0875	[23]
Secondary active case per active case	0.1	0	0.5	Assumption based on national surveillance data and whole-genome sequencing cluster analyses

### Epidemiological data

#### Cascade of care

Cascade of care data were based on empirical data from the study site using the cohort described above and summarized in annex 1 [16].

#### Activation rate

Activation rate of LTBI infection to active TB is higher in the first 2 years of exposure. It was assumed to be 2.5% per year for the first 2 years, and 0.1% per year for all following years [20, 21].

#### Secondary transmission

A fixed rate of 0.1 active secondary case per active case due to secondary transmission was assumed based on Swedish surveillance data including comprehensive whole-genome sequencing and epidemiological information for the period 2016–2018.

#### LTBI treatment efficacy

The treatment efficacy, meaning the reduction of risk of activation, varies in the literature. It was chosen as 77.5% for the 4-month rifampin regimen, which is the most commonly prescribed treatment within this patient group in Stockholm [22].

#### Death due to TB

Persons treated for active TB have increased the yearly risk of death by 7% according to Canadian data, which was assumed to be the same in Stockholm due to the lack of national data [23].

#### Death

Death from general causes has been taken from the official registry of general mortality rates in Sweden [24].

### HRQoL data

Local HRQoL data were collected from persons treated for LTBI [24] and TB [25], respectively, at Karolinska University hospital in the period 2017–2018. These HRQoL decrements were used in the present analysis, meaning that no decrements were associated with the diagnosis or treatment of LTBI group as the analysis showed no statistically significant difference in HRQoL between LTBI patients and Stockholm population [25], while a decrement of 0.28 per year (0.14 per 6 months) was used for patients diagnosed and treated for TB [26]. Neither persons treated for LTBI nor TB patients included in these studies reported any severe adverse drug reactions. Nausea, dizziness and body pain were the main side effects reported [24, 25]

### Cost data

The societal perspective costs included: direct medical costs of screening, treatment, and management (including auxiliary services such as translators); direct non-medical costs including transportation costs for patients and companions; and indirect costs in terms of productivity loss.

Ingredient cost data were derived from different local databases and references summarized in Table 2. For the present screening program, costs were calculated by multiplying ingredient costs with the number of persons in the cohort completing each step in the LTBI screening and treatment cascade, based on empirical cascade data reported elsewhere [16]. Costs included: (1) the cost of screening during the HE, including IGRA and CXR for those doing these tests and 20% of the HE staff cost for all entering the cohort (HE includes several other elements and 20% of staff time was estimated, based on observations, to be attributed to TB screening and related information); (2) cost of subsequent TB clinic visit for those referred and treatment costs for those initiating treatment (doctor visit, nurse visit, medicines, tests, transportation, translator and productivity loss). The no screening scenario was not associated with any LTBI screening or treatment cost.

**Table 2:** Summary of cost data used in the model

Type of cost	Value (SEK)	Reference
Direct medical		
CXR	1008	Karolinska hospital records
IGRA	573	Karolinska hospital records
Tests for TB investigation (no hospitalization)	5978	Karolinska hospital records
Tests for TB investigation (with hospitalization)	50,000	Karolinska hospital records
Nurse visit	1600	Karolinska hospital records
Doctor visit for active TB	4600	Karolinska hospital records
Doctor visit for LTBI	2260	Karolinska hospital records
Liver enzymes test	21	Karolinska hospital records
LTBI treatment cost (by age)		
< 10	1054	
10–18	2108	Karolinska hospital records
> = 18	2121	
TB treatment cost (by age)		
< 10	4931	
10–18	4931	Karolinska hospital records
> = 18	5339	
Health examination cost	2080	SKL*
Hospitalization due to TB	111,000	Karolinska hospital records
Cost of MDR-TB treatment	400,000	Karolinska hospital records and expert opinion
Contact investigation	22,638	Karolinska hospital records
Direct non-medical		
Transportation (1 trip)	45	Stockholm Public transport tariff
Translator (1 h)	394	Karolinska hospital records
Indirect/productivity loss		
Cost per hour	172	Statistics Sweden (SCB)(28)

*Cost is based on a standard compensation of 2080 kronor (2017) from the migration office for executing a health examination. Reference: Sveriges Kommuner och Landsting, Hälso- och sjukvård åt asylsökande under år 2015 [Health care for asylum seekers in 2015]. PM 2016-08-25. Vårt dnr: 16/04,417

Costs (and savings) of treating active TB were calculated based on ingredient costs for active TB, including diagnosis, treatment, management and contact investigation, multiplied by number of modelled incident active TB cases over the 50-year time horizon in each scenario.

Due to difficulties for asylum seekers to enter the Swedish labour market, the monthly cost of productivity loss was calculated based on the lowest 10th percentile monthly salary in 2016, which was 22,000 SEK, plus 31% social fees paid by employer to the state [27, 28]. Therefore, the total monthly productivity loss was 28,912 SEK, divided into 21 workings days with 8 working h/day which leads to a cost of 172 SEK/h. This cost has been added to all ages, as for children it was assumed that one adult will miss work to assist the child.

## Results

The present screening strategy applied during 4 years 2015–2018 was estimated to prevent 25 TB cases over the coming 50 years. The highest number of prevented cases, 18, was in the age group 13–19 (Table 3).

**Table 3:** Results of cost-effectiveness analysis by age groups and country and by TB incidence in country of origin

Age groups (years) disaggregated by incidence in country of origin (per 100 000)	Number of people screened	Total cost, no screening programme (SEK)	Total cost, present screening programme (SEK)	Total incremental cost (SEK)	Total QALY, no screening programme	Total QALY, present screening programme	Total incremental QALY	ICER (SEK/QALY)	Active TB cases, no screening programme	Active TB cases, present screening programme	Incremental cases prevented	ICER (SEK/prevented case)
0–12	**370**	**306,730**	**755,540**	**448**,**810**	**9554.029**	**9554.584**	**0.555**	***808,667***	**2.22**	**1.4097**	**0.8103**	***553,881***
< 50*	66	60,869	142,753	81,884	1704.19	1704.31	0.12	***682,367***	0.44	0.28	0.16	***511,775***
50–100*	56	38,811	104,434	65,623	1446.06	1446.13	0.07	***937,471***	0.27	0.18	0.09	***729,144***
100–199	116	67,102	199,042	131,940	2995.49	2995.6	0.11	***1,199,455***	0.44	0.3	0.14	***942,429***
200–299	40	68,977	128,241	59,264	1032.65	1032.8	0.15	***395,093***	0.53	0.32	0.21	***282,210***
> = 300	92	105,933	226,407	120,474	2375.41	2375.63	0.22	***547,609***	0.78	0.49	0.29	***415,428***
13–19)	**1752**	**4,292,400**	**7,859,472**	**3**,**567**,**072**	**44888.5176**	**44900.256**	**11.7384**	***303,881***	**33.288**	**17.30976**	**15.97824**	***223,246***
< 50*	75	94,390	211,914	117,524	1922.14	1922.38	0.24	***489,683***	0.7	0.38	0.32	***367,263***
50–100*	106	446,932	736,454	289,522	2714.71	2716	1.29	***224,436***	3.53	1.8	1.73	***167 354***
100–199	1355	3,246,784	5,976,854	2,730,070	34717.28	34,726,25	8.97	***304,356***	25.11	13.1	12.01	***227,316***
200–299	164	766,108	1,243,424	477,316	4199.66	4201.89	2.23	***214,043***	6.07	3.08	2.99	***159,637***
> = 300	52	112,769	212,881	100,112	1332.4	1332.7	0.3	***333,707***	0,87	0.46	0.41	***244,176***
***20–34***	**2007**	**6,275,889**	**9,479,061**	**3,203,172**	**50597.0721**	**50601.4875**	**4.4154**	***725,455***	**48.168**	**42.42798**	**5.74002**	***558,042***
< 50*	273	401,139	773,205	372,066	6885.14	6885.4	0.26	***1,431,023***	2.97	2.62	0.35	***1,063,046***
50–100*	355	760,624	1,278,004	517,380	8951.77	8952.29	0.52	***994,962***	5.77	5.07	0.7	***739,114***
100–199	727	1,965,536	3,082,341	1,116 805	18329.76	18331.15	1.39	***803,457***	15.09	13.22	1.87	***597,222***
200–299	314	1,025,100	1,532,197	507,097	7915.78	7916.52	0.74	***685,266***	7.93	6.94	0.99	***512,219***
> = 300	338	1,406,853	1,995,310	588,457	8518.99	8520.01	1.02	***576,919***	10.96	9.58	1.38	***426,418***
***35–54***	**1125**	**4,520,250**	**6,273,000**	**1**,**752**,**750**	**26,059**	**26,060**	**0.79**	**2,225,714**	**32.625**	**31.5**	**1.125**	**1,558,000**
< 50*	139	348,520	549,066	200,546	3220.94	3221	0.06	***3,342,433***	2.51	2.43	0.08	***2,506,825***
50–100*	248	954,802	1,338,145	383,343	5744.88	5745.05	0.17	***2,254,959***	6.99	6.76	0.23	***1,666,709***
100–199	288	1,079,057	1,521,948	442,891	6671,64	6671.83	0.19	***2,331,005***	7.89	7.64	0.25	***1,771,564***
200–299	182	851,081	1,143,936	292,855	4215.17	4215.32	0.15	***1,952,367***	6.26	6.05	0.21	***1,394,548***
> = 300	268	1,280,919	1,714,279	433,360	6206.81	6207.03	0.22	***1,969,818***	9.42	9.12	0.3	***1,444,533***
***55 + ***	**219**	**768,909**	**1,127,412**	**358,503**	**3,577**	**3,578**	**0.09**	**4,092,500**	**4.60**	**4.51**	**0.09**	**4,092,500**
< 50*	27	79,213	121,963	42,750	441.12	441.13	0.01	***4,275,000***	0.48	0.47	0.01	***4,275,000***
50–100*	26	93,347	136,117	42,770	424.72	424.73	0.01	***4,277,000***	0.56	0.55	0.01	***4,277,000***
100–199	99	326,552	486,694	160,142	1617.31	1617.34	0.03	***5,338,067***	1.97	1.92	0.05	***3,202,840***
200–299	41	156 172	224,461	68,289	669.71	669.73	0.02	***3,414,450***	0.94	0.92	0.02	***3,414,450***
> = 300	26	100,932	144,416	43,484	424.69	424.7	0.01	***4,348,400***	0.61	0.60	0.01	***4,348,400***

Table 3 presents the average costs and effects per person and ICER for screening compared to no screening by different age groups. The lowest ICER was in the age group 13–19 years, and this was the only age group with an ICER of less than 500,000 SEK/QALY. ICER was in the range 500,000–1,000,000 SEK/QALY for the age groups 0–12 and 20–34 years, while it was above 1,000,000 for the older age groups. Incremental cost per prevented active TB case followed the same trend as cost per QALY.

Table 3 also summarizes total costs, number of incident cases and total QALYs with and without the current screening program disaggregated by country TB incidence categories within each age group. Screening individuals aged 13–19 had ICER below 500,000 SEK/QALY for all country TB incidence categories. Screening individuals from countries with incidence > 100/100,000 had ICERs < 500,000 SEK/QALY only in the 13–19 age group. In the 20–34 age group, the ICER approached but was still above 500,000 SEK/QALY for screening people from countries with incidence > 300 per 100,000. Screening people over the age of 34 had an ICER > 1,000,000 SEK/QALY regardless of country of origin.

### Sensitivity analysis

**Fig. 2:**
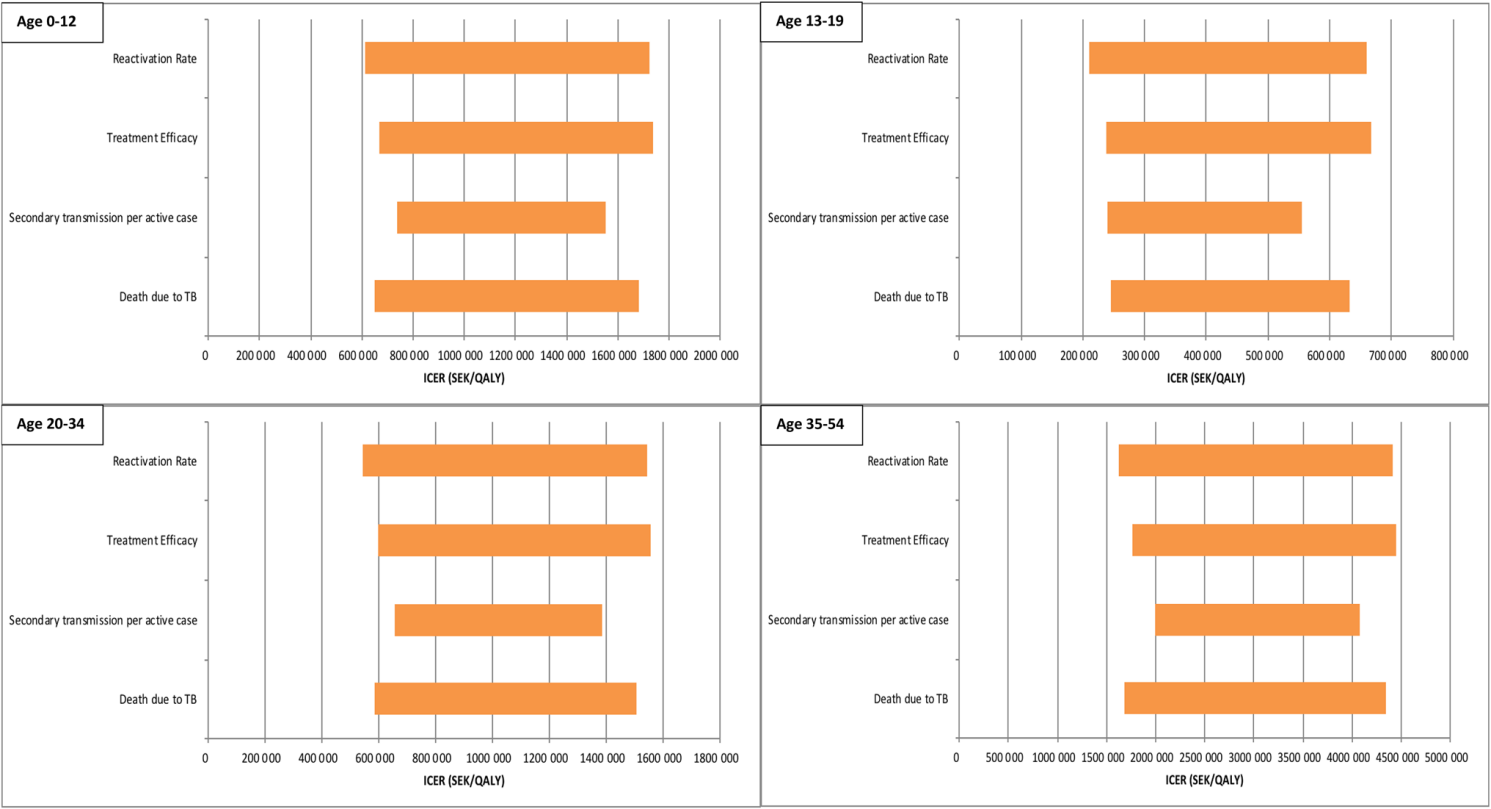
Results of the one-way sensitivity analysis for the base case in different age groups using the upper and lower values of parameters reported in Table 1*. ICER* incremental cost-effectiveness ratio; *SEK* Swedish Krona; *QALY* quality-adjusted life years; *TB* tuberculosis

Figure 2 shows the results of the sensitivity analyses. Although variation in each parameter resulted in large ICER changes, the ICER remained above 500,000 SEK/QALY in all variations for age groups 0–12 and 20–34 and above 1,000,000 SEK/QALY for all variations in those 35 years of age or older.

### Scenario analysis

For the age group 20–34, we ran a scenario analysis to predict the effect of a hypothetical increase in the referral and treatment initiation rates. If the completion of these steps of the cascade would be the same as empirically observed for the age group 13–19 (70% visiting TB clinic, 65% of those starting treatment and 94% of them completing treatment), the incremental cost per prevented case across all countries of origin would be 208,812 SEK and incremental cost per QALY would be 274,626 SEK.

Another scenario analysis was run to predict the effect of IGRA positivity on the economic results. The IGRA positivity value was increased by 25% (relative to each respective base case estimate), which lead to decrease in the ICER value to 662,222, 265,772, 607,993, 1,890,357, and 4 643,264 SEK/QALY in the 5 age groups, respectively. When decreasing the IGRA positivity value by 25%, the ICERs increased to 1,025,329, 357,266, 892,085, 2,870,483, and 7,043,975 SEK/QALY in the 5 age groups, respectively.

## Discussion

To our knowledge, this is the first study assessing the cost-effectiveness of migrant LTBI screening in Sweden. Our model estimates that the implementation of the present screening approach between 2015 and 2018 will prevent 25 TB cases over 50 years.

Cost-effectiveness of LTBI screening is dependent on patient and provider adherence [16]. We have previously shown that the present screening strategy is implemented largely according to policy and that patient adherence is high [15]. Still, this screening strategy is only clearly cost-effective for some subgroups, while it is not cost-effective for others. Despite the lack of absolute cost-effectiveness thresholds to guide health care prioritization in Sweden, the National Board of Health and Welfare usually considers an intervention cost-effective if the ICER is below 500,000 SEK/QALY. Our findings, therefore, suggest that screening individuals in the age group 13–19 is cost-effective and should continue to be recommended. With ICERs in the range of 500,000–1,000,000 SEK/QALY screening in children aged 0–12 and adults aged 19–34 falls in the category of high cost per QALY (“moderately cost effective”), but this is not an absolute reason for not recommending it. The reason for higher ICER in the age group 0–12 than in 13–19 is the lower prevalence of positive IGRA, which means a much small fraction of those screened are candidates for LTBI treatment. However, on the individual level, LTBI treatment may be especially valuable in young children.

Our results show that a change in the present strategy towards referring more LTBI patients in the age group 19–34 to TB clinic and initiating preventive treatment for them could increase the cost-effectiveness within this group. The same conclusion has been drawn concerning LTBI screening in Norway, another Scandinavian country with a similar TB epidemiology and profile of migrants, where the results emphasized the need to increase treatment initiation in IGRA-positive patients below the age of 35 years [29]. Therefore, more access to preventive treatment for this group may be recommended, especially since our local data did not show hepatotoxicity or other major adverse effects during treatment [25]. Treating this age group is in line with international LTBI management guidelines and recommendations [1].

The restrictive policy for LTBI treatment for the age group 20–34 in Stockholm has been questioned and might be seen as conservative compared to other regions of Sweden where treatment is recommended for those up to the age of 35 [15, 29] In addition, the cascade of care data collected in this cohort shows high rates of treatment completion rates [15] which is promising for the effectiveness and cost-effectiveness of LTBI screening. A recent review concluded that effectiveness of LTBI programs in the EU/EEU is largely limited by a weak care cascade when a minority of migrants who are screened complete preventive treatment [30], which seems not to be a problem in the Stockholm setting.

The ICERs for screening persons above the age of 34 was well above the 1,000,000 SEK/QALY threshold regardless of country of origin. The present screening approach in this age group is, therefore, definitely not cost-effective. This is mainly due to the fact that these groups are usually not eligible for LTBI treatment. Preventive treatment is generally not recommended for patients older than 35 in Sweden according to the public health agency guidelines, mainly due to the higher risk of adverse drug reactions that might outweigh the benefit of preventive treatment [29].

The main rationale for IGRA screening in this age group is to detect active TB, as it increases screening sensitivity compared to only screening for TB symptoms [29]. In our analysis, we have accounted for the reduction in secondary cases due to early detection of active TB and the population-level preventive effect is minimal. In the 4-year period we have assessed, only two active TB cases were detected through screening in this age group at HE. It is not known how many of them were detected through symptom screening vs. IGRA screening. Regardless, the added value of IGRA screening is probably very modest as long as preventive treatment is not offered. Whether increasing the age threshold for recommending preventive treatment or discontinuing IGRA screening in this age group and instead replace it with symptom screening only or symptom screening plus CXR requires further analysis.

The cost-effectiveness analysis performed by ECDC in four European countries (Spain, Portugal, Netherlands and Czech Republic) concluded that LTBI screening of migrants at entry is cost-effective when persons from high-incidence countries are targeted, which is in line with our results [10]. In addition, previous reviews and original studies [29–32] suggested that screening young migrants with IGRA is cost-effective, especially those coming from countries with TB incidence > 150/100,000 [32]. However, interpretation and comparisons across settings and studies requires caution due to the methodological differences and the use of different approaches of modelling and assumptions [16].

One of the study strengths is that we have relied on recent local data on epidemiology, costs, cascade of care as well as HRQoL; in addition, our results are robust and supported by the sensitivity analysis with a reasonable level of certainty about the cost-effectiveness of the screening strategy for the age group 13–19. However, assumptions and simplifications such as the modelling of TB states and the costing approach have been made and need to be taken into consideration when interpreting the results. Other limitations are: (1) not directly considering the adverse drug reactions and its consequences in term of HRQoL, especially hepatotoxicity; (2) assuming a 100% sensitivity and specificity of IGRA; (3) assuming the same activation rate for all patients without differentiating in term of age, risk factors or time since infection; (4) assuming a 100% efficacy of TB treatment meaning that all patients that are treated for TB are assumed to return to “healthy” after finishing the treatment.

Another limitation of this analysis is the fluctuation in IGRA positivity rates by incidence rate categories in country of origin. This fluctuation makes it hard to reliably predict cost-effectiveness in different subgroups. Despite not influencing the general recommendations, the sensitivity analysis sheds light on the importance of IGRA positivity as a variable that can influence the results of LTBI screening. In sum, it should be kept in mind that migration patterns vary between and within countries and change over time and along with that the IGRA positivity in different groups. Consequently, the recommendations drawn through this analysis might not apply in another setting or in the future.

## Conclusion

This study assessed incremental costs of LTBI screening among migrants in Stockholm in relation to potential future health benefits and concluded that screening young individuals from high-incidence countries is cost-effective, especially persons in the age group 13–19 years who have recently migrated from a country with an incidence > 100/100,000. Effectiveness and cost-effectiveness of the screening program for patients of age 19–34 years could be enhanced through targeting highest incidence countries and ensuring a high rate of referral and initiation of preventive treatment. For individuals over the age of 34, this study shows that LTBI screening with the current strategy is not cost-effective even for migrants from high-incidence countries.

## Appendix

See Table 4

**Table 4:** Summary of epidemiological parameters used in the economic analysis

Age groups disaggregated by incidence in country of origin	+ IGRA from the cohort**	Chest X-ray referral from the screened cohort ***	Visited TB clinic among IGRA + ^†^	Started treatment among IGRA + ^‡^	Finished treatment when started^§^
0–12	0.05	0.04	0.68	0.55	1
< 50*	0.06				
50–100*	0.04				
100–199	0.03				
200–299	0.13				
> = 300	0.08				
13–19	0.19	0.17	0.7	0.66	0.94
< 50*	0.09				
50–100*	0.35				
100–199	0.19				
200–299	0.39				
> = 300	0.17				
20–34	0.26	0.2	0.31	0.17	0.83
< 50*	0.11				
50–100*	0.17				
100–199	0.22				
200–299	0.27				
> = 300	0.35				
35–54	0.37	0.28	0.19	0.04	0.67
< 50*	0.22				
50–100*	0.35				
100–199	0.22				
200–299	0.43				
> = 300	0.44				
55 +	0.45	0.34	0.15	0.03	1
< 50*	0.37				
50–100*	0.46				
100–199	0.42				
200–299	0.49				
> = 300	0.5				

*Only those with epidemiological risk factors are screened

**Data reported by Nederby-Öhd et al. [15], Table 1

***Data are obtained through the same database explained in Nederby-Öhd et al. [15], however, not reported in the article itself

^†^Values are the product of “Referred to TB specialist clinic of those screened positive by IGRA” and “Visit at TB specialist clinic of those referred” in Nederby-Öhd et al. [15], Table 2

^‡^Data reported by Nederby-Öhd et al. [15], Table 2

^§^Data reported by Nederby-Öhd et al. [15], Table 3
